# Predicting the targeting of tail-anchored proteins to subcellular compartments in mammalian cells

**DOI:** 10.1242/jcs.200204

**Published:** 2017-05-01

**Authors:** Joseph L. Costello, Inês G. Castro, Fátima Camões, Tina A. Schrader, Doug McNeall, Jing Yang, Evdokia-Anastasia Giannopoulou, Sílvia Gomes, Vivian Pogenberg, Nina A. Bonekamp, Daniela Ribeiro, Matthias Wilmanns, Gregory Jedd, Markus Islinger, Michael Schrader

**Affiliations:** 1 Biosciences, University of Exeter, Exeter EX4 4QD, UK; 2 Centre for Cell Biology/Institute of Biomedicine & Department of Biology, University of Aveiro, Aveiro 3810-193, Portugal; 3 Met Office Hadley Centre, Exeter EX1 3PB, UK; 4 Temasek Life Sciences Laboratory, Department of Biological Sciences, National University of Singapore, Singapore; 5 EMBL Hamburg, c/o DESY, Hamburg 22603, Germany; 6 Institute of Neuroanatomy, Center for Biomedicine and Medical Technology Mannheim, University of Heidelberg, Mannheim 68167, Germany

**Keywords:** Tail-anchored protein, Peroxisomes, Mitochondria, ACBD5

## Abstract

Tail-anchored (TA) proteins contain a single transmembrane domain (TMD) at the C-terminus that anchors them to the membranes of organelles where they mediate critical cellular processes. Accordingly, mutations in genes encoding TA proteins have been identified in a number of severe inherited disorders. Despite the importance of correctly targeting a TA protein to its appropriate membrane, the mechanisms and signals involved are not fully understood. In this study, we identify additional peroxisomal TA proteins, discover more proteins that are present on multiple organelles, and reveal that a combination of TMD hydrophobicity and tail charge determines targeting to distinct organelle locations in mammals. Specifically, an increase in tail charge can override a hydrophobic TMD signal and re-direct a protein from the ER to peroxisomes or mitochondria and vice versa. We show that subtle changes in those parameters can shift TA proteins between organelles, explaining why peroxisomes and mitochondria have many of the same TA proteins. This enabled us to associate characteristic physicochemical parameters in TA proteins with particular organelle groups. Using this classification allowed successful prediction of the location of uncharacterized TA proteins for the first time.

## Abstract (precis)

**Summary:** Characteristic physicochemical features of tail-anchored membrane proteins, based on the interplay between transmembrane domain hydrophobicity and tail charge, allow prediction of their subcellular localization.

## INTRODUCTION

Tail-anchored (TA) proteins possess a single transmembrane domain (TMD) close to their C-terminus, which anchors them to cellular membranes and exposes their N-terminal domain to the cytosol. They play key roles in processes requiring membrane anchorage such as organelle division, apoptosis, vesicle targeting/fusion and lipid trafficking (Borgese and Fasana, 2011). Their correct targeting and localization is therefore of fundamental importance for cellular function and viability of the organism. As the TMD of TA proteins emerges from the ribosome only after termination of translation, sorting and insertion require post-translational mechanisms (Borgese and Fasana, 2011; Kutay et al., 1993). TA proteins gain entry to the cellular membrane systems at three subcellular sites: the endoplasmic reticulum (ER), mitochondria and peroxisomes. In yeasts and mammals, the orthologous GET and TRC40 complexes, respectively, are involved in the delivery and insertion of TA proteins into the ER (Mariappan et al., 2010; Mateja et al., 2015; Schuldiner et al., 2008). In mammals initial binding of nascent TA proteins is mediated by the SGTA and the BAG6 complex, constituting a quality control step in the pathway (Hessa et al., 2011; Leznicki and High, 2012; Leznicki et al., 2013; Mariappan et al., 2010; Mock et al., 2015). Following successful transit through the SGTA/BAG6 checkpoint, TA proteins are delivered to the ER transit factor TRC40 (GET3 in yeast). Two additional proteins, WRB (Vilardi and Lorenz, 2011) and CAML (also known as L1CAM) (Yamamoto and Sakisaka, 2012), then act as receptors for TRC40-bound TA proteins on the ER membrane. For some ER TA proteins, alternative pathways exist which may utilize the signal recognition particle (SRP) or HSC70–HSP40 systems (Abell et al., 2004, 2007; Daniele et al., 2016; Vogl et al., 2016). The molecular mechanisms for sorting and insertion to peroxisomes and mitochondria are less clearly understood. The factors for targeting of mitochondrial TA proteins have not yet been identified, although the involvement of HSC70 (also known as HSPA8) has been suggested (Borgese and Fasana, 2011; Rabu et al., 2008). An alternative possibility is via unassisted insertion, with the composition of the mitochondrial membrane contributing to targeting specificity (Kemper et al., 2008). For peroxisomal TA proteins, targeting is generally considered to be mediated by PEX19, an import receptor for peroxisomal membrane proteins (PMPs), and PEX3, the receptor for PEX19-bound PMPs at the peroxisomal membrane (Chen et al., 2014b; Yagita et al., 2013). Owing to the few peroxisomal TA proteins identified to date, these studies are based on mammalian PEX26 (PEX15p in yeast) (Buentzel et al., 2015; Halbach et al., 2006; Yagita et al., 2013) and FIS1, which is present on both peroxisomes and mitochondria (hereafter denoted as shared) (Delille and Schrader, 2008; Koch et al., 2005). Besides these primary targeting systems, subcellular localization can be further controlled after membrane entry by processes such as membrane extraction and TA protein degradation (Chen et al., 2014a; Okreglak and Walter, 2014).

The targeting information for TA proteins is contained within the C-terminus, and it is established that hydrophobicity of the TMD and the presence of charged residues are important factors in membrane selection (Borgese et al., 2007). Generally, ER-targeted TA proteins tend to have more hydrophobic TMDs than those targeted to mitochondria (Wang et al., 2010) with non-charged regions surrounding the TMD (Horie et al., 2002; Kuroda et al., 1998). Yagita and colleagues (2013) demonstrated that, for PEX26, charged residues in the tail were also important for peroxisomal targeting. Despite this general knowledge about factors influencing targeting, it remains to be determined how these two properties ensure proper targeting to mitochondria, peroxisomes and the ER.

Furthermore, TA proteins can be targeted to both peroxisomes and mitochondria in mammalian cells (Dixit et al., 2010; Gandre-Babbe and van der Bliek, 2008; Huber et al., 2013; Koch et al., 2005), revealing close organelle interplay and novel peroxisomal functions (Schrader et al., 2015). Moreover, disorders with combined defects in peroxisomal and mitochondrial fission, caused by mutations in MFF and GDAP1, TA proteins shared by both organelles, have been discovered (Huber et al., 2013; Koch et al., 2016; Shamseldin et al., 2012). As peroxisomes fulfill important metabolic functions in lipid and reactive oxygen species (ROS) metabolism, and influence neuronal development and aging (Fransen et al., 2012), there is great interest in the identification of additional peroxisomal TA proteins and those shared by peroxisomes and mitochondria.

Although bioinformatic studies have previously identified potential TA proteins in yeast, plants and humans (Beilharz et al., 2003; Kalbfleisch et al., 2007; Kriechbaumer et al., 2009; Shigemitsu et al., 2016), wider, integrated studies focusing on how targeting is coordinated to control organelle selection in mammals are currently lacking. Here, we expand the repertoire of peroxisomal TA proteins, reveal additional proteins shared by multiple organelles, and show that a combination of TMD hydrophobicity and tail charge determines targeting to distinct organelles in mammals. We demonstrate that tail charge and TMD hydrophobicity act as directly opposing signaling parameters. A sufficient increase in one can override the other, re-directing a protein from the ER to peroxisomes or mitochondria and vice versa. Mechanistically, changes in these physicochemical parameters correlated with the ability of either PEX19 or GET3 to bind and prevent aggregation of individual TA proteins. We show that subtle alterations in TMD hydrophobicity and tail charge can shift targeting between organelles, explaining why peroxisomes and mitochondria share many TA proteins. Our analyses allow, for the first time, successful prediction of the location of uncharacterized TA proteins.

## RESULTS

### Identification of new peroxisomal and shared peroxisome and mitochondria TA proteins

**Fig. 1.**
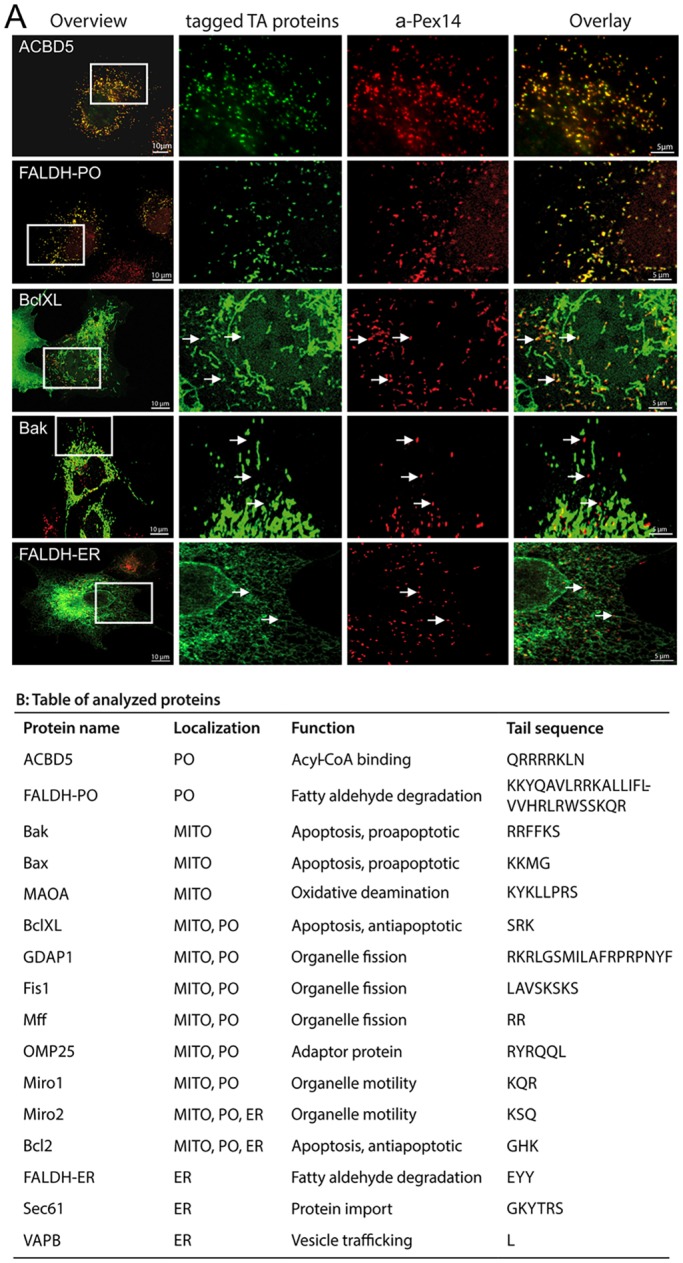
**Targeting survey for TA proteins in mammalian cells.** (A) Subcellular localization patterns for selected TA proteins. COS-7 cells transfected with Myc–ACBD5, Myc–FALDH-PO, Myc–FALDH-ER, GFP–BCL-XL or GFP–BAK were immunolabeled using anti-PEX14 (PO) and anti-Myc antibodies. Arrows highlight regions of colocalization (BCL-XL) or lack of colocalization (BAK, FALDH-ER) with peroxisomes. Higher magnifications of boxed regions are shown. Scale bars: 10 µm (overview), 5 µm (overlay). (B) Table summarizing the TA proteins analyzed. MITO, mitochondria; PO, peroxisomes.

Peroxisomes and mitochondria cooperate in lipid and ROS metabolism and share membrane proteins involved in organelle division and anti-viral signaling (Koch et al., 2005; Delille and Schrader, 2008; Gandre-Babbe and van der Bliek, 2008; Dixit et al., 2010). Remarkably, all these dually localized proteins are TA proteins. To assess how extensive sharing of TA proteins between organelles is, and to identify additional peroxisomal proteins, we examined a number of TA proteins for localization and targeting (Fig. 1; Fig. S1). Expression of tagged TA proteins in COS-7 cells and colocalization with organelle markers revealed a subset of mitochondrial TA proteins that were able to target both mitochondria and peroxisomes. These included the anti-apoptotic proteins BCL-XL (encoded by *BCL2L1*) and BCL2, the motor adaptors MIRO1 and MIRO2 (also known as RHOT1 and RHOT2, respectively), and OMP25 (also known as SYNJ2BP) (Fig. 1; Fig. S1). BCL2 and MIRO2 were additionally targeted to the ER, which has already been reported for BCL2 (Krajewski et al., 1993). Peroxisomal localization of MIRO1 was confirmed by detection of the endogenous protein in organelle subfractions (Fig. S2).

In contrast, the pro-apoptotic TA proteins BAK (also known as BAK1) and BAX were targeted to mitochondria as was monoamine oxidase A (MAOA) (Fig. 1; Fig. S1). Expression of ER TA proteins SEC61β, VAPB and FALDH isoform 2 (denoted FALDH-ER in this study; FALDH is also known as ALDH3A2) (Ashibe et al., 2007) resulted in ER staining (Fig. 1; Fig. S1); for FALDH-ER, localization was confirmed by assessing organelle subfractions (Fig. S2B). FALDH-PO, a splice variant of FALDH which only differs from FALDH-ER in its C-terminal tail (Fig. 1B) (Ashibe et al., 2007) was confirmed as a TA protein that exclusively targets peroxisomes (Fig. 1; Fig. S2E). ACBD5 is another potential TA protein recently detected at peroxisomes (Islinger et al., 2007; Nazarko et al., 2014; Wiese et al., 2007). Overexpressed and endogenous ACBD5 showed peroxisomal localization in COS-7 cells (Fig. 1A; Fig. S2A). Furthermore, ACBD5 localized to peroxisomal fractions separated by density gradient centrifugation and was found in the integral membrane protein fraction after carbonate treatment; differential permeabilization experiments also showed that its N-terminus faces the cytosol (Fig. S2C–E). Overall, these observations suggest that, in addition to TA proteins targeting either mitochondria or peroxisomes, a subset of mitochondrial TA proteins share overlapping targeting properties with peroxisomal TA proteins. In contrast, all tested ER-specific TA proteins showed no detectable peroxisomal localization.

### High TMD hydrophobicity is not unique to ER TA proteins in mammals, but peroxisomal TA proteins contain a highly charged tail

**Fig. 2.**
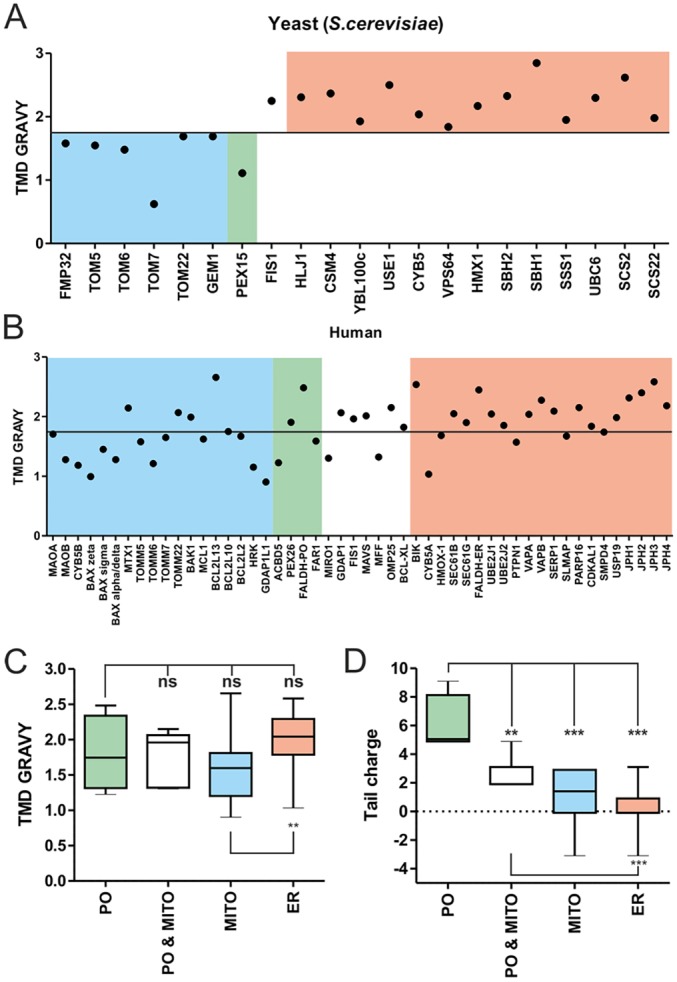
**Comparison of physicochemical parameters of human and yeast TA proteins.** (A–C) Localization of TA proteins in humans (Kalbfleisch et al., 2007) and yeast (Beilharz et al., 2003) was assessed via database and literature searches; TMD GRAVY and net tail charge were calculated for each. (A,B) Scatter plots depicting TMD GRAVY for each TA protein in yeast (A) and humans (B). (C,D) Box-and-whisker plots of tail charge (D) and TMD GRAVY (D) for human TA proteins. The box represents the 25–75th percentiles, and the median is indicated. The whiskers show the sample range. **P<0.01; ***P<0.001; ns, not significant compared to indicated group (unpaired *t*-test). Mitochondria (MITO) TA proteins (blue); peroxisome (PO) TA proteins (green); shared TA proteins (PO and MITO, white); ER TA proteins (salmon pink).

Targeting information responsible for sorting of TA proteins to the ER and mitochondria is generally located within their C-termini (Borgese et al., 2003). Targeting signals are supposed to consist of general physicochemical parameters such as TMD hydrophobicity and tail charge. Compared to ER TA proteins, mitochondrial TA proteins are generally thought to possess less hydrophobic TMDs (Borgese and Fasana, 2011). This is the case for yeast, where ER TA proteins clearly differ from those targeted to mitochondria or peroxisomes by a more hydrophobic TMD (GRAVY>1.75) (Fig. 2A) (Beilharz et al., 2003). To identify organelle-specific targeting information for mammalian TA proteins, we analyzed the C-terminal sequences of 51 proteins whose localization had been characterized (including this study) and compared their physicochemical parameters (Dataset S1 available at https://doi.org/10.6084/m9.figshare.4758532). Whereas in yeast, a clear distinction between ER and mitochondrial TMD hydrophobicity is observed, this does not universally apply to mammalian TA proteins. Here, TMD hydrophobicity is more randomly distributed and not significantly different when compared to peroxisomal TA proteins (Fig. 2A–C). However, our analysis revealed a significantly higher positive net charge of the tail region in peroxisomal TA proteins (6.03±1.03) compared to those routed to both peroxisomes and mitochondria (mean 2.5±0.43), to mitochondria only (mean 1.12±0.41) or to ER (mean 0.21±0.3) (mean±s.e.m., *n*=51; Fig. 2D). Significant differences in charge or hydrophobicity were not observed in regions preceding the TMD (Fig. S3). When tail length was assessed, peroxisomal TA proteins appeared to possess a significantly longer tail (Fig. S3A), but this did not appear to be a requirement for peroxisomal targeting, as ACBD5 contains a short tail comparable to the average tail length of the other groups.

We conclude that a highly positive net charge in the tail is a general property of all identified peroxisomal TA proteins in mammals (as shown for PEX26 by Yagita et al., 2013), which distinguishes them from mitochondrial and ER TA proteins. We further determined that there was a significantly higher TMD hydrophobicity in ER compared to mitochondrial TA proteins, indicating that a hydrophobic TMD and low tail charge support ER targeting.

### Alterations in tail charge and TMD hydrophobicity distribute TA proteins between peroxisomes, mitochondria and ER

**Fig. 3.**
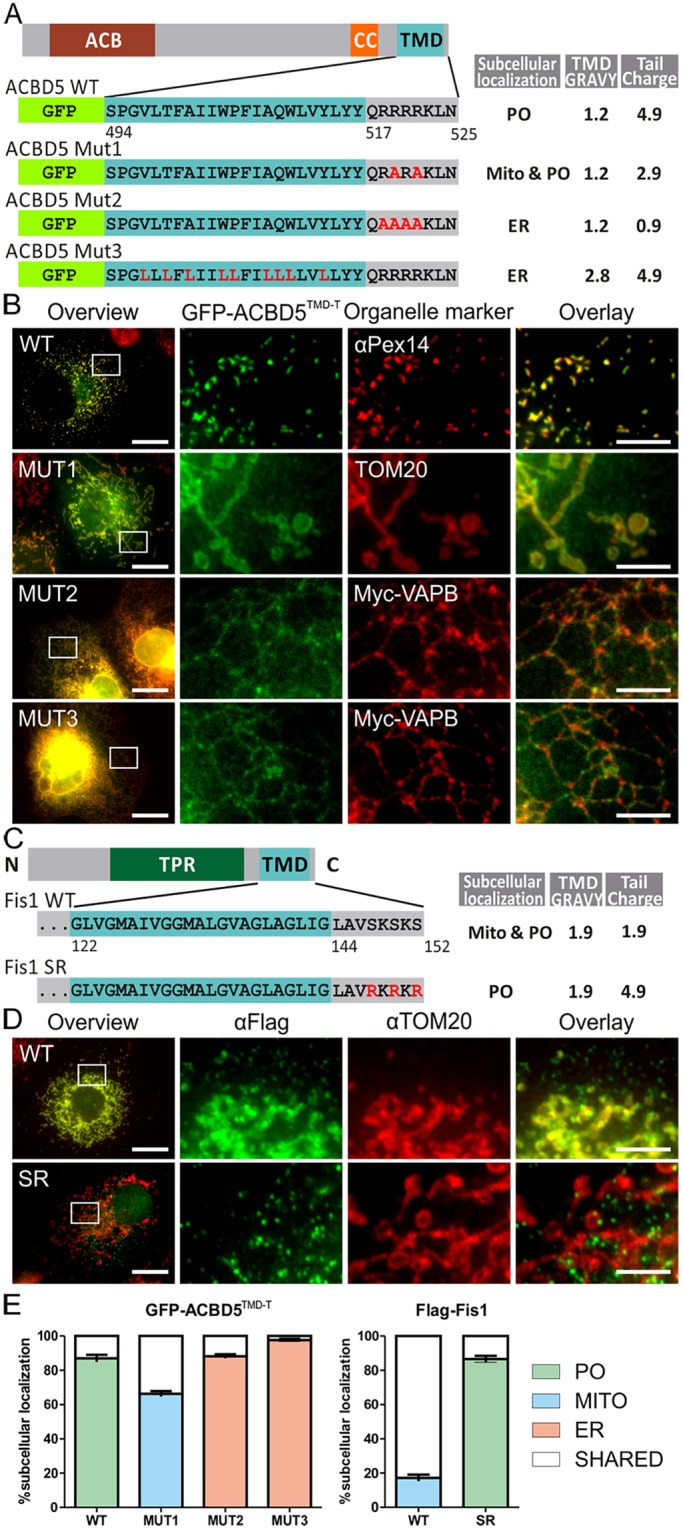
**Alterations in tail charge and TMD GRAVY redistribute TA proteins to other organelles.** (A) Domain structure of ACBD5, and the GFP–ACBD5^TMD-T^ WT and mutant (MUT1–MUT3) constructs used. ACB, acyl CoA-binding domain. (B) COS-7 cells transfected with GFP–ACBD5^TMD-T^ WT or MUT1–MUT3 and, where indicated, Myc–VAPB (ER), were labeled with anti-PEX14 (for peroxisomes, PO), anti-TOM20 (for mitochondria, MITO) and anti-Myc antibodies. (C) Domain structure of FIS1 WT and SR. TPR, tetratricopeptide repeat domain. (D) COS-7 cells transfected with FLAG fusions of FIS1-WT or FIS1-SR were labeled with anti-TOM20 and anti-FLAG antibodies. (E) Qualitative analysis of GFP–ACBD5^TMD-T^ (A,B) and FLAG–FIS1 (C,D) localization. A minimum of 300 cells were examined per condition, and organelle localization was microscopically assessed. The percentage of cells with PO, MITO, ER or shared localization is shown (for ACBD5 WT or MUT1 and FIS1-WT, shared is the percentage of cells with both PO and MITO staining; for ACBD5 MUT2, shared is the percentage of cells with MITO and ER staining; for ACBD5 MUT3, shared is the percentage of cells with ER and PO staining). Values represent mean±s.e.m. of three independent experiments. Higher magnification view of boxed regions in B and D is shown. Scale bars: 20 µm (overview), 10 µm (overlay).

To verify the bioinformatics results, we first analyzed a selection of ACBD5 mutants (Fig. 3A). The GFP-tagged ACBD5 TMD and C-terminal tail (GFP–ACBD5^TMD-T^) fusion protein was targeted to peroxisomes, indicating that the TMD and tail region is sufficient for peroxisomal targeting (Fig. 3B). Mutations in the tail region (GFP–ACBD5^TMD-T^ MUT1), reducing tail charge from +4.9 to +2.9, resulted in targeting to mitochondria (Fig. 3B). Some dual localization to peroxisomes and mitochondria was observed for this mutant (‘shared’ in Fig. 3E), but all cells showed mitochondrial targeting (Fig. 3E; Fig. S4A). Further reducing tail charge to +0.9 (GFP–ACBD5^TMD-T^ MUT2), predominantly resulted in ER staining, with some cells showing targeting to both ER and mitochondria (Fig. 3B,E; Fig S4A). This demonstrates that subtle changes in tail charge can route a peroxisomal TA protein to mitochondria, whereas further reduction in charge leads to ER targeting.

Our analysis showed that some ER TA proteins possess a positively charged tail, but unlike peroxisomal TA proteins this is generally combined with a highly hydrophobic TMD. To investigate whether an increase in TMD hydrophobicity can direct ACBD5 to the ER and ‘override’ the positively charged tail, we expressed a version of GFP–ACBD5^TMD-T^ (MUT3) with increased TMD hydrophobicity (Fig. 3A). MUT3 was directed to the ER and showed only minor peroxisomal targeting (Fig. 3B,E). This is in line with our data on FALDH, which possesses a highly hydrophobic TMD (GRAVY 2.4). This property (and the negative charge in the tail of –1.1) routes the major isoform (FALDH-ER) to the ER (Fig. 1A). Targeting a TA protein with a highly hydrophobic TMD to peroxisomes appears to require a highly positive net charge in the tail. Indeed, the tail of FALDH-PO is highly charged (charge +9.1), and overrides TMD hydrophobicity. To investigate whether increased tail charge can improve targeting to peroxisomes, we expressed a mutant version of FIS1 with increased tail charge, denoted FIS1-SR (Onoue et al., 2013) (Fig. 3C). Wild-type FIS1, as described previously (Koch et al., 2005), distributes to both mitochondria and peroxisomes, whereas FIS1-SR is predominantly targeted to peroxisomes (Fig. 3D,E; Fig S4B).

**Fig. 4.**
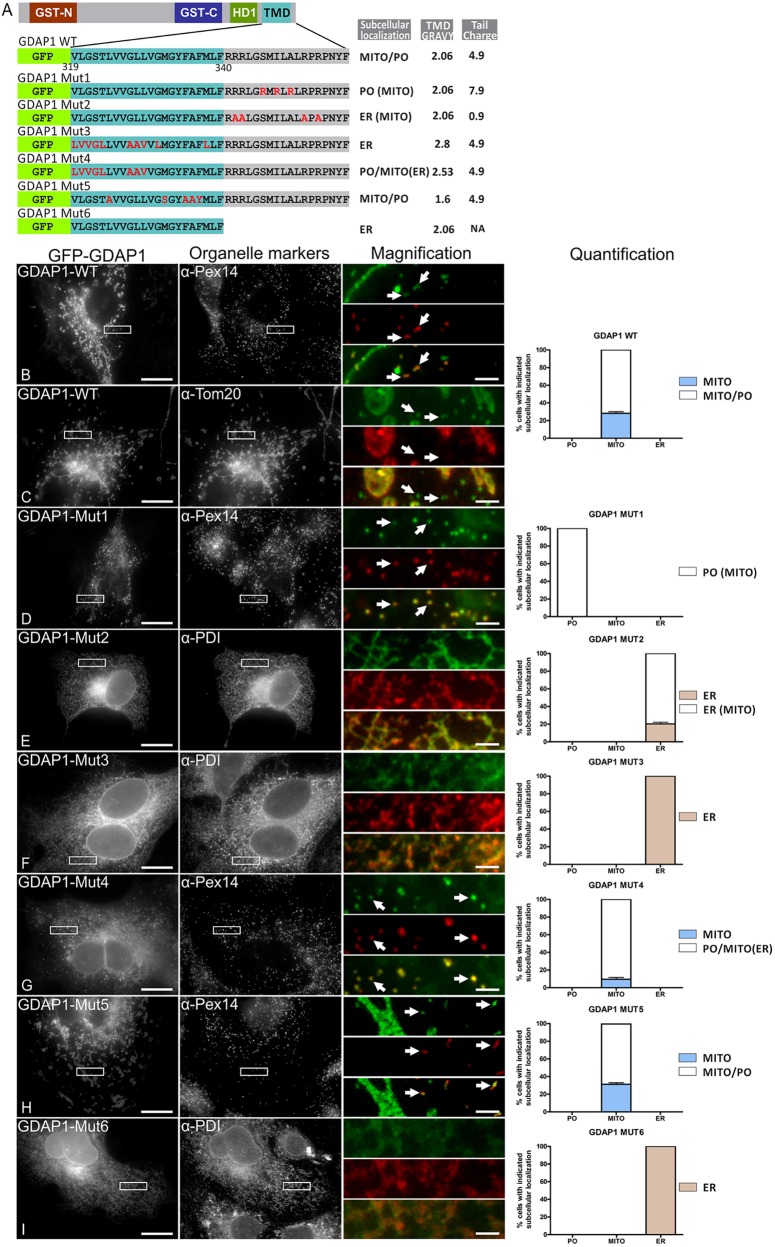
**Alterations in tail charge and TMD GRAVY redistribute GDAP1 to other organelles.** (A) Domain structure of GDAP1, and the GFP–GDAP1^TMD-T^ WT and mutant (MUT1–MUT6) constructs used. (B–I) COS-7 cells were transfected with GFP–GDAP1^TMD-T^ WT or MUT1–MUT6 and labeled with anti-PEX14 (for peroxisomes, PO), anti-TOM20 (for mitochondria, MITO) and anti-PDI (ER) antibodies. Arrows highlight regions of colocalization with PEX14 (B,D,G,H) or lack of colocalization with TOM20 (C). For qualitative assessment, the percentage of cells with PO, MITO, ER or shared (between organelles indicated by ‘/’) localization for the individual constructs is shown. A minimum of 300 cells were examined per condition, and organelle localization was microscopically assessed. An organelle name in parentheses indicates very weak but observable staining. Values represent mean±s.e.m. of three independent experiments. Higher magnification views of boxed regions are shown. Scale bars: 20 µm (main images), 2.5 µm (magnifications).

To more definitively establish correlations between TA protein sequence and localization, we utilized another model TA protein, GDAP1, which is predominantly mitochondrial but also localizes and functions at peroxisomes (Huber et al., 2013). We generated a systematic set of mutants with alterations in both TMD hydrophobicity and tail charge and assessed their localization (Fig. 4). This is shown graphically for each individual mutant in Fig. 4B–I as the percentage of cells displaying the indicated subcellular localization. For example, a GFP–GDAP1^TMD-T^ fusion protein with wild-type TMD and tail sequence was targeted to mitochondria alone in ∼28% of cells whereas ∼72% of cells showed dual mitochondrial and peroxisomal targeting (Fig. 4B,C). In line with our other observations, increasing tail charge increased peroxisomal targeting of GDAP1 (while not completely abolishing mitochondrial targeting) whereas reducing tail charge resulted in ER targeting (Fig. 4D,E). Increasing TMD hydrophobicity was able to override tail charge, resulting in predominantly ER targeting (Fig. 4F) whereas reducing the hydrophobicity caused a shift to mitochondria (Fig. 4G,H). Finally, removing the tail altogether resulted in ER targeting (Fig. 4I). Overall, our data suggest an interplay between tail charge and TMD hydrophobicity in organelle targeting. We conclude that a highly charged tail in combination with a moderately hydrophobic TMD directs TA proteins to peroxisomes. Subtle changes can alter protein distribution: a reduction in tail charge or TMD hydrophobicity enables targeting of peroxisomal TA proteins to mitochondria whereas low charges in combination with a highly hydrophobic TMD favor transport to the ER; an increase in tail charge increases peroxisomal targeting by directly opposing the hydrophobic ER signal in the TMD. Our analysis also reveals that an increase in TMD hydrophobicity can ‘override’ tail charge and route peroxisomal TA proteins to the ER.

### Peroxisomal TA proteins interact with the peroxisomal import receptor PEX19

**Fig. 5.**
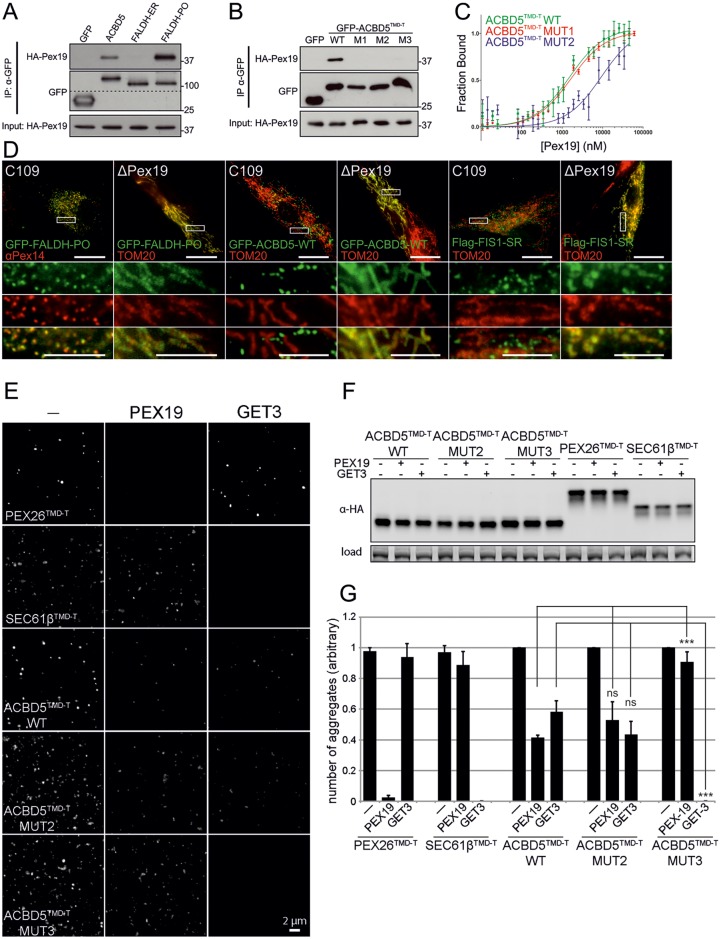
**PEX19 affinity is a key determinant in targeting the peroxisomal membrane.** (A,B) Immunoblots of co-immunoprecipitations from COS-7 cell lysates from cells expressing HA–PEX19 and GFP fusions as indicated, using GFP-Trap. Cytosolic GFP was used as a control. Input (1% of total), total cell lysates; IP, immunoprecipitation. A dotted line in A indicates where a region of gel is not shown for better visualization. (C) Normalized representative curves of fluorescence anisotropy measurements, using recombinant PEX19 and fluorescently labeled peptides (ACBD5-TMD-T) (see Fig. 3A). Average *K*_d_ (µM) values were WT, 0.9±0.5; MUT1, 1.9±0.5; MUT2, 7.7±0.2. Values represent mean±s.d. of three independent measurements. (D) Control and PEX19-deficient fibroblasts transfected with GFP and FLAG fusions as indicated were labeled with anti-PEX14, anti-TOM20 and anti-FLAG antibodies. Higher magnification view of boxed regions is shown. Scale bars: 20 µm (top panel), 5 µm (lower panels). (E) mRNAs for ACBD5^TMD-T^ constructs, PEX26 ^TMD-T^ and SEC61β ^TMD-T^ were *in vitro* translated in the presence of recombinant *Nc*PEX19 or GET3 (5 µM) and aggregation was monitored by using fluorescence microscopy. Scale bar: 2 µm. (F) Immunoblots showing levels of *in vitro*-translated proteins. Equal amounts of a representative translation reaction were loaded and the blot probed with an anti-HA antibody; a band from a Coomassie-stained gel run in parallel serves as a loading control. (G) Solubilizing activity as determined by quantification of aggregate number with data from 10 individual fields of view. Values represent mean±s.e.m. of three independent experiments. ***P<0.001; ns, not significant compared to the indicated group (unpaired *t*-test).

Targeting of membrane proteins to peroxisomes involves the import receptor PEX19 (Sacksteder et al., 2000). Owing to the restricted number of known peroxisomal TA proteins, studies on PEX19 interaction have focused on PEX26 (yeast PEX15p) (Chen et al., 2014b; Halbach et al., 2006; Yagita et al., 2013). For the dually targeted TA proteins FIS1 and GDAP1, interaction with PEX19 has been demonstrated (Delille and Schrader, 2008; Huber et al., 2013). Immunoprecipitation experiments revealed that the peroxisomal TA proteins FALDH-PO and ACBD5 interact with PEX19, whereas no interaction was observed for FALDH-ER (Fig. 5A). As the FALDH isoforms only differ in the tail sequence, this points to a role for the tail in PEX19 binding. Interaction with PEX19 was also demonstrated for FIS1 and FIS1-SR (Fig. S3G). With the GFP–ACBD5^TMD-T^ fusions interaction was observed for the wild type (WT), but not for mutants 1–3 suggesting a requirement of high charge and moderate TMD hydrophobicity for PEX19 binding (Fig. 5B). This was confirmed *in vitro* by testing binding of fluorescently labeled peptides matching the TMD and tail region of ABCD5 to recombinant PEX19 by using fluorescence anisotropy (Fig. 5C). Whereas binding of the WT and MUT2 peptides to PEX19 was significantly different (*K*_d_=0.9 µM and 7.7 µM, respectively), binding of peptide MUT1 to PEX19 was only slightly altered compared to WT (MUT1, *K*_d_=1.9 µM). Binding of the fluorescent peptides to the control peroxisomal protein SurE was not observed, indicating specificity for PEX19 (Fig. S3H). The discrepancy between binding of MUT1 to PEX19 *in vitro* compared with the lack of interaction observed in the immunoprecipitation experiments may reflect the presence of competing factors *in vivo* (see Discussion).

We also investigated the targeting of peroxisomal TA proteins in PEX19-deficient fibroblasts. In control cells, FALDH-PO, ACBD5 and FIS1-SR were targeted to peroxisomes (Fig. 5D), whereas all three proteins were routed to mitochondria in PEX19-deficient cells (Fig. 5D) supporting a general role for PEX19 in receptor-mediated targeting of peroxisomal TA proteins. Importantly, all three proteins showed no observable ER localization in PEX19-deficient cells (Fig. S3), further confirming the overlap between peroxisomal and mitochondrial-targeting properties. Mechanistic insights into the biochemical activity of *Neurospora crassa* (*Nc*) PEX19 and the ER-targeting factor GET3 have recently been revealed using a cell-free assay (Chen et al., 2014b). By using this assay, the authors demonstrated that *Nc*PEX19 but not *Nc*GET3 was sufficient to prevent aggregation of *Nc*PEX26. To gain further insight into the mechanisms controlling selective organelle targeting we utilized this assay to test the activity of PEX19 and GET3 on our ACBD5^TMD-T^ constructs. ACBD5^TMD-T^ constructs were *in vitro* translated in HeLa extracts in the presence of recombinant *Nc*PEX19 or *Nc*GET3. *Nc*PEX26^TMD-T^ and *Nc*SEC61β^TMD-T^ served as controls. In the absence of PEX19 and GET3, TA proteins form large aggregates seen as punctate structures in fluorescence microscopy images (Fig. 5E). Translation in the presence of PEX19 largely prevented aggregation of ACBD5^TMD-T^ WT, and ACBD5^TMD-T^ MUT2 but had almost no effect on ACBD5^TMD-T^ MUT3 (comparable effect to that seen with SEC61β). By contrast, GET3 prevented aggregation of MUT3, but had significantly less impact on MUT2 and WT. Artificial aggregation by saturation of the chaperoning machinery was excluded by the use of excess *Nc*PEX19 and *Nc*GET3. For ACBD5^TMD-T^ MUT3 this correlates with our localization and PEX19-binding data, suggesting that upon increasing TMD hydrophobicity PEX19 activity is reduced whereas for GET3 it is apparently increased. However, a charged tail does not exclude GET3 from having some activity on ACBD5^TMD-T^ WT *in vitro*, in line with previous observations (Yagita et al., 2013). Nevertheless, the positive charge in the tail of ACBD5^TMD-T^ WT increases the binding affinity to PEX19 when compared to an uncharged tail sequence, as shown by the fluorescence anisotropy assay (Fig. 5C).

### Prediction of TA protein localization in mammalian cells

**Fig. 6.**
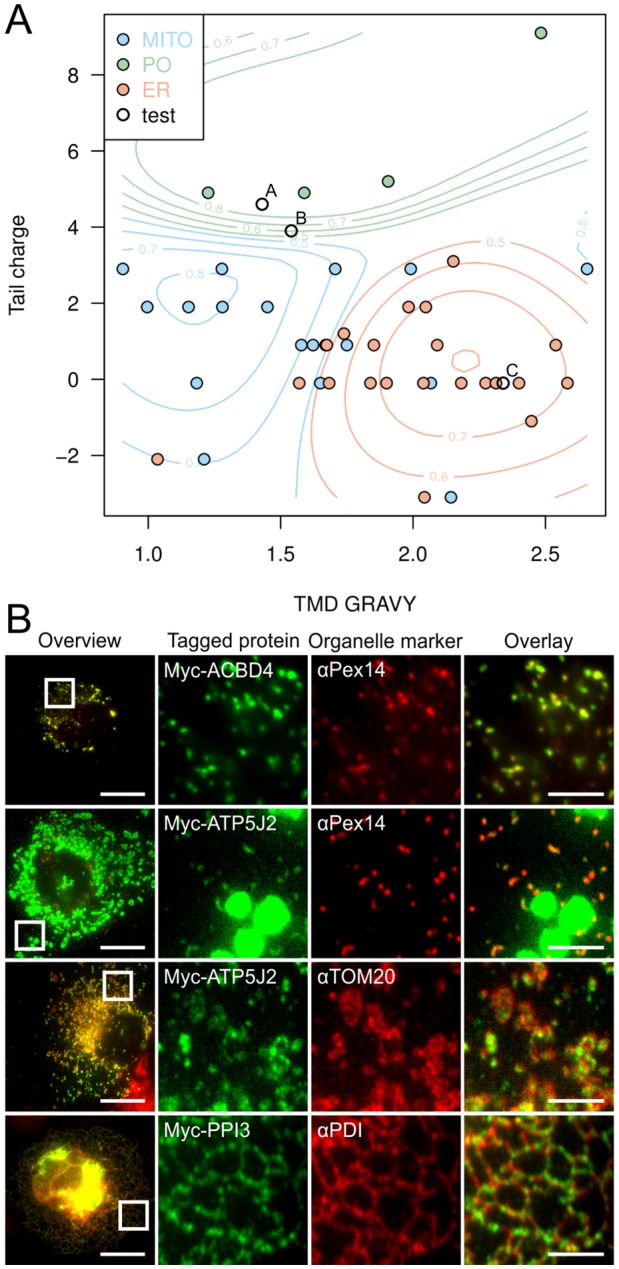
**A combination of tail charge and TMD GRAVY allows prediction of organelle targeting for mammalian TA proteins.** (A) SVM classifier plot showing clustering of TA proteins to different organelle locations based on TMD GRAVY and tail charge. Probability contours are as indicated. Test represents selected TA proteins: A, ACBD4; B, ATP5J2; C, PPP1R3F. (B) COS-7 cells transfected with Myc fusions of ACBD4, ATP5J2, and PPP1R3F were labeled with anti-PEX14, anti-TOM20, anti-PDI (ER) and anti-Myc antibodies. Higher magnification views of boxed regions are shown. Scale bars: 20 µm (overview), 10 µm (overlay).

Finally, we exploited the compiled data to predict targeting of uncharacterized TA proteins (Fig. 6). We trained a SVM classifier using the TMD GRAVY, tail charge and cellular location of 43 proteins from our dataset (Fig. 1 and Dataset S1 available at https://doi.org/10.6084/m9.figshare.4758532). This classifier builds a statistical model able to predict the probability of a protein to be targeted to each organelle (Fig. 6A). Peroxisomal, mitochondrial and ER TA proteins can clearly be separated into regions of high-class probability or clusters, with very few exceptions (i.e. mitochondrial TOMM22 clusters with ER). When using the highest probability class, the SVM misclassifies 9 of the 43 data points (21%) when used in an in-sample fashion. A more rigorous leave-one-out cross validation misclassifies 14 of the 43 data points (33%). To assess the predictive power of our classifier, we analyzed a published list of predicted human TA proteins (Kalbfleisch et al., 2007) and generated probabilities for peroxisomal, mitochondrial and ER targeting (Dataset S2 available at https://figshare.com/s/07df2992d588a2f0c98d). The predicted localization of three proteins was experimentally verified (Fig. 6B). In agreement with our prediction, the candidate TA protein ACBD4 localized to peroxisomes (Fig. 6B). ACBD4 shares 58% sequence identity with ACBD5, mainly due to similarities in the N-terminal acyl-CoA-binding domain, but the amino acid sequence in the C-terminus is significantly different. ATP5J2, which was shown to be a minor component of the mitochondrial ATP synthase complex (Aggeler et al., 2002), was also predicted to be targeted to peroxisomes. Expression of Myc–ATP5J2 revealed dual targeting to mitochondria and peroxisomes (Fig. 6B). This is in accordance with proteomics studies reporting other ATP synthase subunits in peroxisomal fractions (Wiese et al., 2007), but how these proteins might function at peroxisomes is unclear. Finally, we analyzed the targeting of PPP1R3F, a potential regulatory subunit of protein phosphatase type 1 complexes (Kelsall et al., 2011). Predicted targeting to the ER was confirmed by expression of Myc–PPP1R3F in COS-7 cells (Fig. 6B).

## DISCUSSION

Hundreds of TA proteins have been predicted bioinformatically in a wide range of organisms (Beilharz et al., 2003; Kalbfleisch et al., 2007; Kriechbaumer et al., 2009), several have been associated with human disorders, but many are still of unknown function or localization. A better understanding of the mechanisms that determine targeting and localization is of great value for the study of TA proteins and organelle function, in particular in humans where mistargeting may cause hitherto undetected disorders.

In the present study, we characterize the physicochemical parameters of a large number of TA proteins in mammals and increase the number of bona fide peroxisomal TA proteins significantly, allowing us to identify targeting information and bioinformatically predict targeting.

**Fig. 7.**
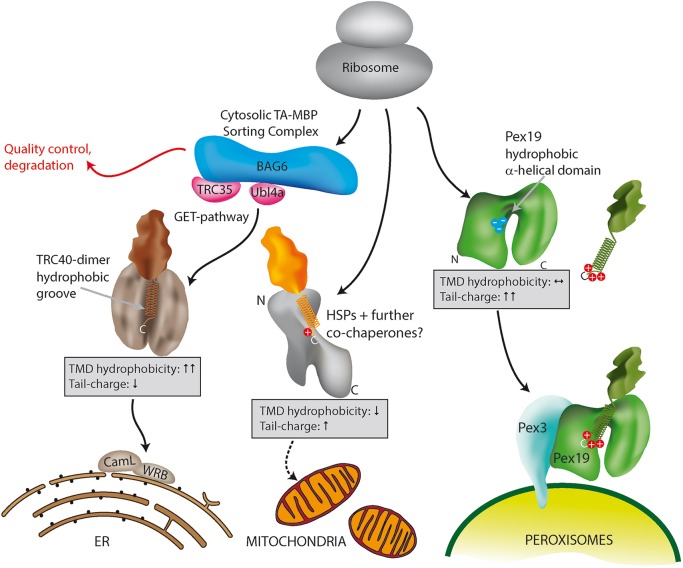
**Schematic model for TA protein targeting to ER, mitochondria and peroxisomes in mammalian cells.** Specific targeting of TA proteins to ER, mitochondria and peroxisomes in mammalian cells is mediated by a combination of TMD hydrophobicity and tail charge. Targeting of TA proteins to the ER involves the GET (guided entry of TA proteins) pathway. ER TA proteins interact with a cytosolic sorting complex (composed of BAG6, TRC35/GET4 and Ubl4a/GET5) and are delivered and inserted into the ER membrane by TRC40 (GET3) and WRB (GET1). A WRB/CAML dimeric membrane receptor (functional homolog to GET1/2) accepts the TA protein from TRC40 at the ER. A hydrophobic TMD and low tail charge support ER targeting in mammals. Targeting of TA proteins to peroxisomes is mediated by PEX19 and PEX3. Peroxisomal TA proteins are characterized by a highly charged tail that promotes PEX19 interaction. TA proteins with a hydrophobic TMD require increased tail charge to be targeted to peroxisomes. It is currently unknown whether delivery and insertion of TA proteins into mitochondria involves specific targeting factors or is primarily unassisted. Mitochondrial TA proteins generally possess a less hydrophobic TMD than ER TA proteins and a less charged tail compared to peroxisomal TA proteins. This scheme is based on the steady state distribution of TA proteins, but other processes such as membrane extraction and TA protein degradation may also influence the subcellular localization. (Please note that the illustration of the GET pathway has been simplified). BAG6, BCL2-associated athanogene cochaperone 6; TRC, transmembrane domain recognition complex; Ubl4a, ubiquitin-like 4a; WRB, tryptophan-rich basic protein.

Recent studies determining targeting properties for mitochondrial and peroxisomal TA proteins demonstrated that targeting to both organelles requires a positively charged C-terminal tail sequence (Horie et al., 2002; Isenmann et al., 1998; Kuroda et al., 1998; Yagita et al., 2013). Our data clearly demonstrate that a highly positive net charge in the tail region is a general property of all identified peroxisomal TA proteins in mammals, which distinguishes them significantly from mitochondrial and ER TA proteins. As shown for ACBD5, a step-wise reduction in the tail charge results first in mitochondrial and subsequently in ER mistargeting. In line with this, an increase in tail charge can direct TA proteins from mitochondria or the ER to peroxisomes, as exemplified by GDAP1 MUT1, FIS1-SR and FALDH-PO. These data fit a model where a highly charged tail promotes interaction with the peroxisome import receptor PEX19 (Fig. 7). We provide evidence that ACBD5, FALDH-PO and FIS1 interact with PEX19, whereas mutants with a reduction in tail charge lose this ability and are mistargeted. This is also reflected in our *in vitro* binding assay using C-terminal peptides. Binding to PEX19 has also been demonstrated for other TA proteins (PEX26, FIS1, GDAP1 and FAR1) (Delille and Schrader, 2008; Halbach et al., 2006; Honsho et al., 2013; Huber et al., 2013). Overall, these findings support a general role for PEX19 in the direct receptor-mediated targeting of peroxisomal TA proteins in mammals (Fig. 7). Nevertheless, additional proteins at the organelle membranes may prevent insertion or induce excision of TA proteins missorted by the cytosolic shuttle systems (Chen et al., 2014a; Okreglak and Walter, 2014).

Whereas in yeast a clear distinction between ER and mitochondrial TMD hydrophobicity is observed, this property does not universally apply to mammalian TA proteins. Instead, our data reveal an interplay between tail charge and TMD hydrophobicity. This is exemplified by FALDH-PO and FALDH-ER, which share a highly hydrophobic TMD, suggesting ER targeting of both. Instead, the highly charged tail routes FALDH-PO to peroxisomes. Our analysis also reveals that an increase in TMD hydrophobicity can ‘override’ tail charge and route peroxisomal TA proteins to the ER and vice versa.

We further determined a significantly higher TMD hydrophobicity in ER TA proteins than in those targeted to mitochondria, indicating that a hydrophobic TMD and a low tail charge support ER targeting in mammals. While this study was under review Rao et al., (2016) proposed helical content of the TMD (based on the AGADIR helix propensity scale) as an additional factor relevant for ER-targeting in yeast. Interestingly, our GDAP1^TMD-T^ model proteins exhibit similar variations in AGADIR values as the model proteins used by Rao and colleagues (GDAP1^TMD-T^ MUT3=1.05, MUT4=0.73, WT=0.57, MUT5=0.48) and similarly show a shift in subcellular localization from the ER to mitochondria. Thus, helical content of the TMD may also be considered as a parameter relevant for TA protein targeting in mammals.

Mitochondrial TA proteins have been proposed to be targeted by a default route allowing either unassisted insertion of TA proteins (Krumpe et al., 2012; Setoguchi et al., 2006) or using as yet uncharacterized targeting factors for mitochondrial TA proteins (Kemper et al., 2008) (Fig. 7). Our data supports that highly hydrophilic TMDs are preferentially inserted into mitochondrial outer membranes (Fig. 3). Interestingly, in the absence of PEX19, a hydrophobic TMD (FALDH-PO) combined with a highly charged tail does not prevent TA protein targeting to mitochondria, and in these conditions no ER localization is observed. This supports a model where mitochondrial TA proteins are targeted by a positive selection mechanism (Fig. 7), suggesting the existence of an, as-yet-undetected shuttle protein, or a default mitochondrial pathway able to insert charged TA proteins with a higher kinetic efficiency than the GET pathway even when they possess highly hydrophobic TMDs. Interestingly, hydrophobic TMD versions of PEX26 and ACBD5 are protected from aggregation by *Nc*GET3 despite their highly charged tails (Chen et al., 2014b; Fig. 5, this study). In their recent publication Rao and colleagues elegantly showed that tail-charge and TMD hydrophobicity influence the yeast GET pathway at three distinct steps: (1) capture by SGT2, (2) transfer from SGT2 to GET3, and (3) targeting and insertion into the ER membrane (Rao et al., 2016). During any of these steps TA proteins may be rejected and would then be available for other organelle-targeting machinery (e.g. PEX19). In the first step, binding to SGT2 depends on the properties of the TMD with no dependence on tail charge. TA proteins with TMDs containing highly hydrophobic or highly helical content form more stable complexes with SGT2 and more readily enter the ER pathway. In the second step, GET3 appears to have similar substrate preferences to SGT2. In the final step GET3-bound substrates that are highly hydrophobic are more likely to be maintained in a stable complex long enough to reach the ER membrane for insertion. At this stage, any positive charge in the tail region drastically reduces import into the ER membrane. Although this model is based on yeast proteins it may provide an explanation for many of the observations we make here. For example, the TMD of ACBD5 has relatively low hydrophobicity/helical content. Thus, based on its TMD, wild-type ACBD5 is a suboptimal substrate for SGT2/GET3. In addition, its charged tail (+4.9, which is higher than the charge observed in any known ER-resident TA protein) would be highly inefficient at inserting into the ER membrane. This would allow PEX19 multiple opportunities to interact with ACBD5 and facilitate its delivery to peroxisomes. When the tail charge is reduced, as in ACBD5^TMD-T^ MUT1, SGT2/GET3 affinity is unchanged, and the tail charge (+2.9) is still suboptimal for ER insertion, but PEX19 affinity is slightly reduced (*K*_d_=1.9 µM vs 0.9 µM in wild-type); potentially the affinity for either a mitochondrial chaperone or the mitochondrial membrane itself is optimal. ACBD5^TMD-T^ MUT2, like MUT1, is able to initially bind to SGT2/GET3 to enter the ER pathway (Fig. 5G) but unlike MUT1 it now has an uncharged tail and so can successfully pass the ER membrane checkpoint.

Our findings also explain why peroxisomes and mitochondria share a significant number of TA proteins such as FIS1, MFF, MAVS and GDAP1 (Koch et al., 2005; Gandre-Babbe and van der Bliek, 2008; Dixit et al., 2010; Huber et al., 2013). Although our results from overexpression experiments cannot definitively prove the *in vivo* localization of all the mitochondrial TA proteins investigated in this study, they still underline the overlap in targeting information for both organelles. As subtle changes in the tail charge can shift TA targeting between peroxisomes and mitochondria, it is likely that some exchange occurred through mutations during co-evolution of both organelles (Martin, 2010). Binding to PEX19 may have been the selective force allowing the development of new functions for peroxisomes. Based on our findings, those shared functions may also include regulation of organelle motility and apoptosis, but these await further confirmation *in vivo*. Very recently peroxisome permeability was reported to be influenced by pro-apoptotic proteins (Hosoi et al., 2017). Thus, anti-apoptotic proteins at peroxisomal membranes could protect the organelles from excessive matrix protein release into the cytosol. Exchange of TA proteins between peroxisomes and the ER appears to be more difficult to achieve, requiring more significant sequence changes. This is exemplified by FALDH, which exploits alternative splicing to allow targeting to peroxisomes or the ER. Here, we demonstrate that the characteristic physicochemical features of the TMD and tail region allow prediction of TA protein localization. Correlating data from the classifier analysis with our experimental approaches confirms that ER TA proteins are primarily sorted according to their high TMD hydrophobicity, which is required for efficient GET3/TRC40 chaperone activity. Peroxisomal TA proteins possess some tolerance in TMD hydrophobicity, but a highly positive tail charge appears to be the primary selective force for PEX19 binding. In mitochondrial TA proteins, low TMD hydrophobicity seems to be favorable for efficient membrane insertion, whereas tail charge appears to shield from selection for effective ER-membrane insertion (Rao et al., 2016). Importantly, both parameters – TMD hydrophobicity and tail charge – exhibit competitive effects on organellar targeting; thus, proteins with a comparatively low or high TMD hydrophobicity, which on its own would favor mitochondrial and ER targeting, respectively, can still be targeted to peroxisomes if the TMD is followed by a highly charged tail. Alternatively, TA proteins with charged tails can be routed to the ER or mitochondria, if they exhibit appropriately hydrophobic or hydrophilic TMDs.

However, besides these general features, other properties may influence organelle-specific targeting, for example additional signals within the N-terminus or accessibility of the tail region. An example may be GDAP1L1, which, when expressed, is cytosolic and can only be targeted to mitochondria upon specific stimulation (Niemann et al., 2014). Additional parameters influencing targeting (e.g. position of hydrophobic and charged residues in the tail, or helical propensity of the TMD) could add another dimension to the classifier, improving its predictive power.

It should be noted that several of the shared peroxisome–mitochondria or peroxisomal TA proteins are of medical importance and have been linked to human disorders (Abu-Safieh et al., 2013; Ferdinandusse et al., 2016; Huber et al., 2013; Keller et al., 2014; Koch et al., 2016; Shamseldin et al., 2012). Our predictor has allowed us to determine potentially new membrane-associated functions for peroxisomes and other organelles. It will be a great challenge for future studies to verify the localization of the endogenous TA proteins, their cell type- or organ-specific expression and to elucidate their cellular functions and importance for organelle biology and human health.

## MATERIALS AND METHODS

### Plasmids and antibodies

For initial cloning of human genes, total RNA was extracted from HepG2 cells by using TRIzol reagent, and was then reverse transcribed into cDNA and used as a PCR template. Gene synthesis was performed by Genscript (Genscript, Piscataway) or Eurofins (Eurofins Genomics, Ebersberg, Germany). See Table S1 for details of plasmids generated in this study, Table S2 for details of primers used and Table S3 for other plasmids. Site-directed mutagenesis was achieved by means of the QuikChange Kit (Agilent). Details on antibodies can be found in Table S4.

### Cell culture and transfection

COS-7 (African green monkey kidney cells; ATCC CRL-1651), HepG2 (human hepatoblastoma cells; ATCC HB-8065), PEX19-deficient (c.320delA) (Mohamed et al., 2010) and wild-type human, control (C109) fibroblasts (kindly provided by Hans Waterham, AMC, University of Amsterdam, The Netherlands) were cultured in DMEM, with high glucose (4.5 g/l) and supplemented with 10% fetal bovine serum (FBS), penicillin and streptomycin at 37°C with 5% CO_2_ and 95% humidity. COS-7 cells were transfected using diethylaminoethyl (DEAE)-dextran (Sigma-Aldrich) or TurboFect™ (Thermo Fisher Scientific). Fibroblasts were transfected by microporation using the Neon^®^ Transfection System (Thermo Fisher Scientific).

### Immunofluorescence and microscopy

Cells were processed for immunofluorescence at 24 or 48 h after transfection. Cells grown on glass coverslips were fixed with 4% paraformaldehyde (PFA) in PBS (pH 7.4), permeabilized with 0.2% Triton X-100 and incubated with antibodies as described previously (Bonekamp et al., 2013). For differential permeabilization, cells were either permeabilized with 0.2% Triton X-100 or 2.5 μg/ml digitonin. Cell imaging was performed using an Olympus IX81 microscope equipped with an UPlanSApo 100×1.40 NA Oil objective (Olympus Optical, Hamburg, Germany). Digital images were taken with a CoolSNAP HQ2 CCD camera and adjusted for contrast and brightness using the Olympus Soft Imaging Viewer software (Olympus Soft Imaging Solutions GmbH) and MetaMorph 7 (Molecular Devices). Confocal images were obtained using a Zeiss LSM 510 META inverted microscope equipped with a Plan Apochromat 63×1.4 NA (oil/dic) objective (Carl Zeiss, Oberkochen, Germany), using the Ar 488 nm and He 543 nm laser lines. Digital images were adjusted for contrast and brightness using the Zeiss LSM Image Browser software (Carl Zeiss MircroImaging GmbH).

### Subcellular fractionation

Peroxisome purification from rat liver was performed as described previously (Islinger et al., 2012). In brief, liver tissue was homogenized in homogenization buffer (HB; 250 mM sucrose, 5 mM MOPS, 1 mM EDTA, 2 mM PMSF, 1 mM DTT, 1 mM ɛ-aminocaproic acid and 0.1% ethanol, pH 7.4) using an Potter–Elvehjem tissue grinder (1 stroke/120 s). The homogenate was clarified in an initial centrifugation step at 600 ***g*** for 10 min. The resulting pellet was re-homogenized and re-centrifuged applying the same conditions; both supernatants were pooled and comprise the post nuclear supernatant (PNS). Subsequently, PNS was centrifuged at 1900 ***g*** for 15 min to yield the pellet of heavy mitochondria (HM). The resulting supernatant was centrifuged at 25,500 ***g*** for 20 min resulting in the light mitochondrial pellet (LM). The corresponding supernatant was again centrifuged at 100,000 ***g*** for 30 min to separate the microsomal pellet (MIC) from cytosol (CYT). To increase the purity of the fractions, each pellet recovered was washed in 5 ml HB/g liver tissue and centrifuged using the same parameters. Highly purified peroxisomes were obtained from the LM pellet by applying a sigmoidal Optiprep gradient from 1.26–1.12 g/ml in a vertical type rotor at an integrated force of 1256×10^6^ ***g*** min. Here, peroxisomes form a distinct band at 1.20 g/ml.

Subcellular separation of homogenates from HepG2 cells was performed in a modified procedure. Harvested cells were homogenized in HB using a syringe (needle 27G, 7 strokes). The differential centrifugation series was performed at 500 ***g*** (PNS), 2000 ***g*** (HM), 20,000 ***g*** (LM), 100,000 ***g*** (MIC and CYT). The LM fraction was subsequently separated on a linear Nycodenz gradient from 1.26–1.12 g/ml at 100,000 ***g*** for 3 h. The gradient was eluted in 12 equal-sized fractions for further analysis.

Integral membrane proteins were prepared from the peroxisome-enriched fraction LM using the carbonate-stripping method (Fujiki et al., 1982). An aliquot of LM was pelleted at 25,500 ***g*** and suspended in a hypo-osmotic TVBE-buffer for organelle rupture (1 mM NaHCO_3_, 1 mM EDTA, 0.01% Triton X-100, pH 7.6). After 30 min incubation on ice the organelle suspension was centrifuged at 100,000 ***g*** to yield a soluble matrix fraction and crude membrane pellet. The membrane pellet was subsequently resuspended in 0.1 M Na_2_CO_3_ and incubated on ice for 30 min to remove peripherally attached membrane proteins. The integral membrane pellet was prepared by centrifugation at 100,000 ***g*** and washed in TVBE buffer applying the same centrifugation parameters. Samples (equal amounts of protein) were subsequently analyzed by immunoblotting.

### Immunoprecipitation

For immunoprecipitation experiments, GFP- or FLAG-tagged TA proteins and HA-tagged PEX19 were expressed in COS-7 cells. After 48 h, cells were washed in PBS and then incubated with 1 mM DSP followed by quenching with 100 mM Tris-HCl pH 7.4. Cells were lysed in ice-cold lysis buffer (25 mM Tris-HCl pH 7.5, 150 mM NaCl, 0.5% Triton X-100, 1 mM PMSF and protease inhibitor cocktail), undissolved material was pelleted by centrifugation at 15,000 ***g*** and lysates mixed with GFP-TRAP (ChromoTek) or FLAG-antibody-coupled agarose beads and incubated for 2 h at 4°C. Beads were subsequently washed extensively with lysis buffer and bound proteins eluted with either Laemmli buffer (GFP-TRAP) or 50 mM NaOH (FLAG beads). Immunoprecipitates and total lysates were subsequently analyzed by immunoblotting.

### Expression and purification of PEX19 and SurE

Full-length human PEX19 was cloned into vector pETM11. For PEX19 expression, *E. coli* BL21(DE3)RIL cells were incubated in autoinduction medium (Studier, 2005) at 20°C for 16 h. Subsequently, cells were pelleted, re-suspended in lysis buffer (50 mM Hepes pH 7.5, 200 mM NaCl, 20 mM imidazole), lysed by sonication, loaded onto Ni-NTA resin and eluted with elution buffer (50 mM Hepes pH 7.5, 200 mM NaCl, 300 mM imidazole). The eluted protein was dialyzed overnight into dialysis buffer (50 mM Tris-HCl pH 7.5, 250 mM NaCl, 0.5 mM TCEP) and simultaneously digested with TEV protease (1:50 molar ratio). The protease, affinity tag and undigested protein were removed via a second affinity chromatography step and the cleaved protein was concentrated and purified via Size-Exclusion Chromatography (HiLoad 16/600 Superdex 75 pg, GE Healthcare). SurE (http://www.uniprot.org/uniprot/Q8LAM2) was expressed as an N-terminal poly-histidine fusion in *E. coli* strain BL21(DE3)RIL using auto-induction medium at 37°C for 4 h and 21°C overnight. Purification was as for PEX19 but following elution the eluted protein was further purified by using a 16/600 Superdex 200 pg column.

### Fluorescence anisotropy

Fluorescently labeled peptides ACBD5 WT (FITC–SPGVLTFAIIWPFIAQWLVYLYYQRRRRKL), MUT1 (FITC–SPGVLTFAIIWPFIAQWLVYLYYQRARAKL) and MUT2 (FITC–SPGVLTFAIIWPFIAQWLVYLYYQAAAAKL) (Genscript) were used in the assay at a final concentration of 6.7 nM. Note that the C-terminal asparagine residue was removed to facilitate peptide synthesis. Assays were performed in black 96-well plates (Greiner) with an Infinite M1000 plate reader (TECAN) regulated at 25°C, with excitation and detection at 470 and 530 nm, respectively. The experiment was performed in dialysis buffer with 0.67 mg/ml BSA to prevent unspecific binding on the surface of the plastic well. The protein concentration series was obtained by successive dilution by a factor of 1.5 and each point was measured in triplicate. Being highly hydrophobic, the peptides have a tendency to aggregate, resulting in an unusual decrease in anisotropy upon protein titration. Addition of detergents in the buffer prevented aggregation, but interfered with the interaction, and therefore we chose to perform the experiment in a detergent-free buffer, which provided reproducible data. Three independent measurements were performed and binding data were analyzed using Prism (GraphPad software). Binding profiles were fitted using a simple model (hyperbolic equation) assuming 1:1 stoichiometry.

### Cell-free chaperone assay

mRNA was generated and purified following the manufacturer's instructions (mMessage mMachine T7 Transcription kit and MEGAclear kit; Ambion). Translation reactions were conducted as previously described (Chen et al., 2014b). In short, mRNAs were translated in HeLa cell translation extracts using the 1-Step Human Coupled IVT Kit for DNA (Thermo Scientific) according to the manufacturer's instructions. Reactions were incubated for 2.5 h using 5 μM of chaperone proteins and western blotting used to control for levels of protein expression. Images of aggregates were taken with an epi-fluorescence microscope (BX51;Olympus) equipped with a 100×1.4 NA oil immersion objective and a GFP filter cube. The excitation wavelength is between 457 and 487 nm, the emission wavelength is between 502 and 538 nm, and the dichroic cut-off wavelength is 495 nm. A total of ten separated images were used to generate a maximum projection image in Fiji software. A magnified representative area is shown in Fig. 5. Aggregates were quantified with the ‘Analyze Particle’ function in Fiji. Three independent experiments were conducted and analyzed with unpaired *t*-test. Purification of *Nc*PEX19 and *Nc*GET3 were performed as described previously (Chen et al., 2014b). Briefly, *Nc*PEX19 and *Nc*GET3 were expressed from the pET15b (Novagen) vector in *E. coli* BL21 (DE3, Stratagene) and purified with Ni-NTA resin following the manufacturer's instructions (Qiagen). Eluted proteins were further purified by size-exclusion chromatography using a Hi-load 16/60 Superdex 200 prep grade column (GE Healthcare) equilibrated in Buffer H (20 mM HEPES, pH 6.8, 50 mM KOAc, 200 mM sorbitol and 1 mM MgCl_2_).

### Sequence and bioinformatics analysis

Data on human TA proteins was sourced from the literature (references in Dataset S1 available at https://figshare.com/s/07df2992d588a2f0c98d). SNARE proteins were omitted as they have been previously shown to differ significantly from other ER TA proteins (Kalbfleisch et al., 2007). Protein sequences were obtained from the NCBI database (http://www.ncbi.nlm.nih.gov/), all isoforms were analyzed and those that lacked a C-terminal TMD were removed. Yeast TA proteins were sourced from literature and by homology with human proteins. For the detection of the membrane-spanning helices in the TA proteins, the TMHMM server v. 2.0 (Krogh et al., 2001) was used. When no TMD was predicted but the protein had been characterized as a TA protein, the TMPred server from ExPASy was used, with a threshold score of 1500 (Hofmann and Stoffel, 1993). As a measure for hydrophobicity, the Grand Average of Hydropathicity (GRAVY) of membrane-spanning helices was calculated (Kyte and Doolittle, 1982), using the ProtParam server from ExPASy (Gasteiger et al., 2005). The charge of the tail sequence was calculated using the Protein Calculator v3.4 (http://protcalc.sourceforge.net). Box-and-whisker plots were created with GraphPad Prism 5 (GraphPad Software) with whiskers representing the smallest and largest value in the sample. PEX19-binding sites were analyzed using the BLOCKS algorithm from the PeroxisomeDB 2.0 database (Schlüter et al., 2007).

For the support vector machine (SVM) classifier (Cortes and Vapnik, 1995), we trained a SVM classifier with the [protein data] using the SVM application in package e1071 (Meyer et al., 2014), of the R statistical programming environment (R Core team, 2014; http://www.R-project.org/) utilizing the LIBSVM library of Chang and Lin (Chang and Lin, 2011). The SVM takes the training set of [Tail Charge, GRAVY and location in cell], and builds a statistical model to predict the probability of [location in cell], given any combination of [Tail Charge, GRAVY]. Initially, we restrict the training data to three unique classes, corresponding to [location in cell] of mitochondria (MITO), peroxisomes (PO) and endoplasmic reticulum (ER).

### Statistical analyses

Analysis of GRAVY, charge, tail length and PEX19 binding were performed using GraphPad Prism 5 software. A two-tailed unpaired *t*-test was used to determine statistical differences against the indicated group (*P<0.05, **P<0.01, ***P<0.001). For qualitative analyses of organelle-specific targeting of TA proteins, a minimum of 300 cells were examined per condition, and organelle localization was microscopically assessed in at least three independent experiments. Data are presented as mean±s.e.m.

## References

[JCS200204C1] Abell B. M., Pool M. R., Schlenker O., Sinning I. and High S. (2004). Signal recognition particle mediates post-translational targeting in eukaryotes. *EMBO J.* 23, 2755-2764. 10.1038/sj.emboj.7600281 15229647 PMC514945

[JCS200204C2] Abell B. M., Rabu C., Leznicki P., Young J. C. and High S. (2007). Post-translational integration of tail-anchored proteins is facilitated by defined molecular chaperones. *J. Cell Sci.* 120, 1743-1751. 10.1242/jcs.002410 17456552

[JCS200204C3] Abu-Safieh L., Alrashed M., Anazi S., Alkuraya H., Khan A. O., Al-Owain M., Al-Zahrani J., Al-Abdi L., Hashem M., Al-Tarimi S. et al. (2013). Autozygome-guided exome sequencing in retinal dystrophy patients reveals pathogenetic mutations and novel candidate disease genes. *Genome Res.* 23, 236-247. 10.1101/gr.144105.112 23105016 PMC3561865

[JCS200204C4] Aggeler R., Coons J., Taylor S. W., Ghosh S. S., García J. J., Capaldi R. A. and Marusich M. F. (2002). A functionally active human F1F0 ATPase can be purified by immunocapture from heart tissue and fibroblast cell lines. Subunit structure and activity studies. *J. Biol. Chem.* 277, 33906-33912. 10.1074/jbc.M204538200 12110673

[JCS200204C5] Ashibe B., Hirai T., Higashi K., Sekimizu K. and Motojima K. (2007). Dual subcellular localization in the endoplasmic reticulum and peroxisomes and a vital role in protecting against oxidative stress of fatty aldehyde dehydrogenase are achieved by alternative splicing. *J. Biol. Chem.* 282, 20763-20773. 10.1074/jbc.M611853200 17510064

[JCS200204C6] Beilharz T., Egan B., Silver P. A., Hofmann K. and Lithgow T. (2003). Bipartite signals mediate subcellular targeting of tail-anchored membrane proteins in Saccharomyces cerevisiae. *J. Biol. Chem.* 278, 8219-8223. 10.1074/jbc.M212725200 12514182

[JCS200204C7] Bonekamp N. A., Islinger M., Lázaro M. G. and Schrader M. (2013). Cytochemical detection of peroxiomes and mitochondria. *Methods Mol. Biol.* 931, 467-482. 10.1007/978-1-62703-056-4_24 23027018

[JCS200204C8] Borgese N. and Fasana E. (2011). Targeting pathways of C-tail-anchored proteins. *Biochim. Biophys. Acta* 1808, 937-946. 10.1016/j.bbamem.2010.07.010 20646998

[JCS200204C9] Borgese N., Colombo S. and Pedrazzini E. (2003). The tale of tail-anchored proteins: coming from the cytosol and looking for a membrane. *J. Cell Biol.* 161, 1013-1019. 10.1083/jcb.200303069 12821639 PMC2173004

[JCS200204C10] Borgese N., Brambillasca S. and Colombo S. (2007). How tails guide tail-anchored proteins to their destinations. *Curr. Opin. Cell Biol.* 19, 368-375. 10.1016/j.ceb.2007.04.019 17629691

[JCS200204C11] Buentzel J., Vilardi F., Lotz-Havla A., Gärtner J. and Thoms S. (2015). Conserved targeting information in mammalian and fungal peroxisomal tail-anchored proteins. *Sci. Rep.* 5, 17420 10.1038/srep17420 26627908 PMC4667187

[JCS200204C12] Chang C.-C. and Lin C.-J. (2011). LIBSVM: a library for support vector machines. *ACM Trans. Intell. Syst. Technol.* 2, 1-39. 10.1145/1961189.1961199

[JCS200204C13] Chen Y.-C., Umanah G. K. E., Dephoure N., Andrabi S. A., Gygi S. P., Dawson T. M., Dawson V. L. and Rutter J. (2014a). Msp 1 / ATAD1 maintains mitochondrial function by facilitating the degradation of mislocalized tail-anchored proteins *EMBO. J.* 33, 1548-1568. 10.15252/embj.201487943 24843043 PMC4198051

[JCS200204C14] Chen Y., Pieuchot L., Loh R. A., Yang J., Kari T. M. A., Wong J. Y. and Jedd G. (2014b). Hydrophobic handoff for direct delivery of peroxisome tail-anchored proteins. *Nat. Commun.* 5, 5790 10.1038/ncomms6790 25517356

[JCS200204C15] Cortes C. and Vapnik V. (1995). Support-vector networks. *Mach. Learn.* 20, 273-297. 10.1007/BF00994018

[JCS200204C16] Daniele L. L., Emran F., Lobo G. P., Gaivin R. J. and Perkins B. D. (2016). Mutation of wrb, a component of the guided entry of Tail-Anchored protein pathway, disrupts photoreceptor synapse structure and function. *Investig. Ophthalmol. Vis. Sci.* 57, 2942-2954. 10.1167/iovs.15-18996 27273592 PMC4898200

[JCS200204C17] Delille H. K. and Schrader M. (2008). Targeting of hFis1 to peroxisomes is mediated by Pex19p. *J. Biol. Chem.* 283, 31107-31115. 10.1074/jbc.M803332200 18782765 PMC2662177

[JCS200204C18] Dixit E., Boulant S., Zhang Y., Lee A. S. Y., Odendall C., Shum B., Hacohen N., Chen Z. J., Whelan S. P., Fransen M. et al. (2010). Peroxisomes are signaling platforms for antiviral innate immunity. *Cell* 141, 668-681. 10.1016/j.cell.2010.04.018 20451243 PMC3670185

[JCS200204C19] Ferdinandusse S., Falkenberg K., Koster J., Mooyer P., Jones R., van Roermund C., Pizzino A., Schrader M., Wanders R., Vanderver A. et al. (2016). ACBD5 deficiency causes a defect in peroxisomal very long-chain fatty acid metabolism. *J. Med. Genet*. [EPub] doi:10.1136/jmedgenet-2016-104132 10.1136/jmedgenet-2016-104132 27799409

[JCS200204C20] Fransen M., Nordgren M., Wang B. and Apanasets O. (2012). Role of peroxisomes in ROS/RNS-metabolism: Implications for human disease. *Biochim. Biophys. Acta* 1822, 1363-1373. 10.1016/j.bbadis.2011.12.001 22178243

[JCS200204C21] Fujiki Y., Hubbard A. L., Fowler S. and Lazarow P. B. (1982). Isolation of intracellular membranes by means of sodium carbonate treatment: application to endoplasmic reticulum. *J. Cell Biol.* 93, 97-102. 10.1083/jcb.93.1.97 7068762 PMC2112113

[JCS200204C22] Gandre-Babbe S. and van der Bliek A. M. (2008). The novel tail-anchored membrane protein Mff controls mitochondrial and peroxisomal fission in mammalian cells. *Mol. Biol. Cell* 19, 2402-2412. 10.1091/mbc.E07-12-1287 18353969 PMC2397315

[JCS200204C23] Gasteiger E., Hoogland C., Gattiker A., Duvaud S., Wilkins M. R., Appel R. D. and Bairoch A. (2005). Protein identification and analysis tools on the ExPASy server. *Proteomics Protoc. Handb.* 571-607. 10.1385/1-59259-890-0:571

[JCS200204C24] Halbach A., Landgraf C., Lorenzen S., Rosenkranz K., Volkmer-Engert R., Erdmann R. and Rottensteiner H. (2006). Targeting of the tail-anchored peroxisomal membrane proteins PEX26 and PEX15 occurs through C-terminal PEX19-binding sites. *J. Cell Sci.* 119, 2508-2517. 10.1242/jcs.02979 16763195

[JCS200204C25] Hessa T., Sharma A., Mariappan M., Eshleman H. D., Gutierrez E. and Hegde R. S. (2011). Protein targeting and degradation are coupled for elimination of mislocalized proteins. *Nature* 475, 394-397. 10.1038/nature10181 21743475 PMC3150218

[JCS200204C26] Hofmann K. and Stoffel W. (1993). TMBASE-a database of membrane spanning protein segments. *Biol. Chem. HoppeSeyler* 374, 166.

[JCS200204C27] Honsho M., Asaoku S., Fukumoto K. and Fujiki Y. (2013). Topogenesis and homeostasis of fatty acyl-CoA reductase 1. *J. Biol. Chem.* 288, 34588-34598. 10.1074/jbc.M113.498345 24108123 PMC3843072

[JCS200204C28] Horie C., Suzuki H., Sakaguchi M. and Mihara K. (2002). Characterization of signal that directs C-tail–anchored proteins to mammalian mitochondrial outer membrane. *Mol. Biol. Cell* 13, 1615-1625. 10.1091/mbc.01-12-0570 12006657 PMC111131

[JCS200204C29a] Hosoi K. I., Miyata N., Mukai S., Furuki S., Okumoto K., Cheng E. H. and Fujiki Y. (2017). The VDAC2-BAK axis regulates peroxisomal membrane permeability. *J. Cell Biol.* 216, 709-722. 10.1083/jcb.201605002 28174205 PMC5350511

[JCS200204C29] Huber N., Guimaraes S., Schrader M., Suter U. and Niemann A. (2013). Charcot-Marie-Tooth disease-associated mutants of GDAP1 dissociate its roles in peroxisomal and mitochondrial fission. *EMBO Rep.* 14, 545-552. 10.1038/embor.2013.56 23628762 PMC3674444

[JCS200204C30] Isenmann S., Khew-Goodall Y., Gamble J., Vadas M. and Wattenberg B. W. (1998). A splice-isoform of vesicle-associated membrane protein-1 (VAMP-1) contains a mitochondrial targeting signal. *Mol. Biol. Cell* 9, 1649-1660. 10.1091/mbc.9.7.1649 9658161 PMC25402

[JCS200204C31] Islinger M., Lüers G. H., Li K. W., Loos M. and Völkl A. (2007). Rat liver peroxisomes after fibrate treatment. A survey using quantitative mass spectrometry. *J. Biol. Chem.* 282, 23055-23069. 10.1074/jbc.M610910200 17522052

[JCS200204C32] Islinger M., Abdolzade-bavil A., Liebler S., Weber G. and Völkl A. (2012). Assessing heterogeneity of peroxisomes: isolation of two subpopulations from rat liver. In *Liver Proteomics: Methods and Protocols* (ed. Josic D. and Hixson D. C.), pp. 83-96. Totowa, NJ: Humana Press. 10.1007/978-1-61779-959-4_6 22903710

[JCS200204C33] Kalbfleisch T., Cambon A. and Wattenberg B. W. (2007). A bioinformatics approach to identifying tail-anchored proteins in the human genome. *Traffic* 8, 1687-1694. 10.1111/j.1600-0854.2007.00661.x 17892534

[JCS200204C34] Keller M. A., Zander U., Fuchs J. E., Kreutz C., Watschinger K., Mueller T., Golderer G., Liedl K. R., Ralser M., Kräutler B. et al. (2014). A gatekeeper helix determines the substrate specificity of Sj{ö}gren-Larsson Syndrome enzyme fatty aldehyde dehydrogenase. *Nat. Commun.* 5, 4439 10.1038/ncomms5439 25047030 PMC4109017

[JCS200204C35] Kelsall I. R., Voss M., Munro S., Cuthbertson D. J. R. and Cohen P. T. W. (2011). R3F, a novel membrane-associated glycogen targeting subunit of protein phosphatase 1 regulates glycogen synthase in astrocytoma cells in response to glucose and extracellular signals. *J. Neurochem.* 118, 596-610. 10.1111/j.1471-4159.2011.07345.x 21668450

[JCS200204C36] Kemper C., Habib S. J., Engl G., Heckmeyer P., Dimmer K. S. and Rapaport D. (2008). Integration of tail-anchored proteins into the mitochondrial outer membrane does not require any known import components. *J. Cell Sci.* 121, 1990-1998. 10.1242/jcs.024034 18495843

[JCS200204C37] Koch A., Yoon Y., Bonekamp N. A., Mcniven M. A. and Schrader M. (2005). A role for fis1 in both mitochondrial and peroxisomal fission in mammalian cells. *Mol. Biol. Cell* 16, 5077-5086. 10.1091/mbc.E05-02-0159 16107562 PMC1266408

[JCS200204C38] Koch J., Feichtinger R. G., Freisinger P., Pies M., Schrödl F., Iuso A., Sperl W., Mayr J. A., Prokisch H. and Haack T. B. (2016). Disturbed mitochondrial and peroxisomal dynamics due to loss of MFF causes Leigh-like encephalopathy, optic atrophy and peripheral neuropathy. *J. Med. Genet.* 4, 270-278. 10.1136/jmedgenet-2015-103500 26783368

[JCS200204C39] Krajewski S., Tanaka S., Takayama S., Schibler M. J., Fenton W. and Reed J. C. (1993). Investigation of the subcellular distribution of the bcl-2 oncoprotein: residence in the nuclear envelope, endoplasmic reticulum, and outer mitochondrial membranes. *Cancer Res.* 53, 4701-4714. 8402648

[JCS200204C40] Kriechbaumer V., Shaw R., Mukherjee J., Bowsher C. G., Harrison A.-M. and Abell B. M. (2009). Subcellular distribution of tail-anchored proteins in Arabidopsis. *Traffic* 10, 1753-1764. 10.1111/j.1600-0854.2009.00991.x 19843281

[JCS200204C41] Krogh A., Larsson B., von Heijne G. and Sonnhammer E. L. L. (2001). Predicting transmembrane protein topology with a hidden Markov model: application to complete genomes. *J. Mol. Biol.* 305, 567-580. 10.1006/jmbi.2000.4315 11152613

[JCS200204C42] Krumpe K., Frumkin I., Herzig Y., Rimon N., Özbalci C., Brügger B., Rapaport D. and Schuldiner M. (2012). Ergosterol content specifies targeting of tail-anchored proteins to mitochondrial outer membranes. *Mol. Biol. Cell* 23, 3927-3935. 10.1091/mbc.E11-12-0994 22918956 PMC3469509

[JCS200204C43] Kuroda R., Ikenoue T., Honsho M., Tsujimoto S., Mitoma J. Y. and Ito A. (1998). Charged amine acids at the carboxyl-terminal portions determine the intracellular locations of two isoforms of cytochrome b5. *J. Biol. Chem.* 273, 31097-31102. 10.1074/jbc.273.47.31097 9813010

[JCS200204C44] Kutay U., Hartmann E. and Rapoport T. A. (1993). A class of membrane proteins with a C-terminal anchor. *Trends Cell Biol.*. 3, 72-75. 10.1016/0962-8924(93)90066-A 14731773

[JCS200204C45] Kyte J. and Doolittle R. F. (1982). A simple method for displaying the hydropathic character of a protein. *J. Mol. Biol.* 157, 105-132. 10.1016/0022-2836(82)90515-0 7108955

[JCS200204C47] Leznicki P. and High S. (2012). SGTA antagonizes BAG6-mediated protein triage. *Proc. Natl. Acad. Sci. USA* 109, 19214-19219. 10.1073/pnas.1209997109 23129660 PMC3511132

[JCS200204C48] Leznicki P., Roebuck Q. P., Wunderley L., Clancy A., Krysztofinska E. M., Isaacson R. L., Warwicker J., Schwappach B. and High S. (2013). The association of BAG6 with SGTA and tail-anchored proteins. *PLoS ONE* 8, e59590 10.1371/journal.pone.0059590 23533635 PMC3606182

[JCS200204C49] Mariappan M., Li X., Stefanovic S., Sharma A., Mateja A., Keenan R. J. and Hegde R. S. (2010). A ribosome-associating factor chaperones tail-anchored membrane proteins. *Nature* 466, 1120-1124. 10.1038/nature09296 20676083 PMC2928861

[JCS200204C51] Martin W. (2010). Evolutionary origins of metabolic compartmentalization in eukaryotes. *Philos. Trans. R. Soc. Lond. B. Biol. Sci.* 365, 847-855. 10.1098/rstb.2009.0252 20124349 PMC2817231

[JCS200204C52] Mateja A., Paduch M., Chang H.-Y., Szydlowska A., Kossiakoff A. A., Hegde R. S. and Keenan R. J. (2015). Structure of the Get3 targeting factor in complex with its membrane protein cargo. *Science* 347, 1152-1155. 10.1126/science.1261671 25745174 PMC4413028

[JCS200204C53] Meyer D., Dimitriadou E., Hornik K., Weingessel A. and Leisch F. (2014). Misc functions of the Department of Statistics (e1071), TU Wien. *R Packag. version 1.6-2* http://cran.r–project.org/package=e1071.

[JCS200204C54] Mock J.-Y., Chartron J. W., Zaslaver M., Xu Y., Ye Y. and Clemons W. M. (2015). Bag6 complex contains a minimal tail-anchor-targeting module and a mock BAG domain. *Proc. Natl. Acad. Sci. USA* 112, 106-111. 10.1073/pnas.1402745112 25535373 PMC4291651

[JCS200204C55] Mohamed S., El-Meleagy E., Nasr A., Ebberink M. S., Wanders R. J. A. and Waterham H. R. (2010). A mutation in PEX19 causes a severe clinical phenotype in a patient with peroxisomal biogenesis disorder. *Am. J. Med. Genet. A* 152, 2318-2321. 10.1002/ajmg.a.33560 20683989

[JCS200204C56] Nazarko T. Y., Ozeki K., Till A., Ramakrishnan G., Lotfi P., Yan M. and Subramani S. (2014). Peroxisomal Atg37 binds Atg30 or palmitoyl-CoA to regulate phagophore formation during pexophagy. *J. Cell Biol.* 204, 541-557. 10.1083/jcb.201307050 24535825 PMC3926955

[JCS200204C57] Niemann A., Huber N., Wagner K. M., Somandin C., Horn M., Lebrun-Julien F., Angst B., Pereira J. A., Halfter H., Welzl H. et al. (2014). The Gdap1 knockout mouse mechanistically links redox control to Charcot-Marie-tooth disease. *Brain* 137, 668-682. 10.1093/brain/awt371 24480485 PMC3927703

[JCS200204C58] Okreglak V. and Walter P. (2014). The conserved AAA-ATPase Msp1 confers organelle specificity to tail-anchored proteins. *Proc. Natl. Acad. Sci. USA* 111, 8019-8024. 10.1073/pnas.1405755111 24821790 PMC4050615

[JCS200204C59] Onoue K., Jofuku A., Ban-Ishihara R., Ishihara T., Maeda M., Koshiba T., Itoh T., Fukuda M., Otera H., Oka T. et al. (2013). Fis1 acts as a mitochondrial recruitment factor for TBC1D15 that is involved in regulation of mitochondrial morphology. *J. Cell Sci.* 126, 176-185. 10.1242/jcs.111211 23077178

[JCS200204C60] Rabu C., Wipf P., Brodsky J. L. and High S. (2008). A precursor-specific role for Hsp40/Hsc70 during tail-anchored protein integration at the endoplasmic reticulum. *J. Biol. Chem.* 283, 27504-27513. 10.1074/jbc.M804591200 18667436 PMC2562055

[JCS200204C61] Rao M., Okreglak V., Chio U. S., Cho H., Walter P. and Shan S. (2016). Multiple selection filters ensure accurate tail-anchored membrane protein targeting. *Elife* 5, e21301 10.7554/elife.21301 27925580 PMC5214336

[JCS200204C62] Sacksteder K. A., Jones J. M., South S. T., Li X., Liu Y. and Gould S. J. (2000). PEX19 binds multiple peroxisomal membrane proteins, is predominantly cytoplasmic, and is required for peroxisome membrane synthesis. *J. Cell Biol.* 148, 931-944. 10.1083/jcb.148.5.931 10704444 PMC2174547

[JCS200204C63] Schlüter A., Fourcade S., Domènech-Estévez E., Gabaldón T., Huerta-Cepas J., Berthommier G., Ripp R., Wanders R. J. A., Poch O. and Pujol A. (2007). PeroxisomeDB: a database for the peroxisomal proteome, functional genomics and disease. *Nucleic Acids Res.* 35, D815-D822. 10.1093/nar/gkl935 17135190 PMC1747181

[JCS200204C64] Schrader M., Costello J., Godinho L. F. and Islinger M. (2015). Peroxisome-mitochondria interplay and disease. *J. Inherit. Metab. Dis.* 38, 681-702. 10.1007/s10545-015-9819-7 25687155

[JCS200204C65] Schuldiner M., Metz J., Schmid V., Denic V., Rakwalska M., Schmitt H. D., Schwappach B. and Weissman J. S. (2008). The GET complex mediates insertion of tail-anchored proteins into the ER membrane. *Cell* 134, 634-645. 10.1016/j.cell.2008.06.025 18724936 PMC2572727

[JCS200204C66] Setoguchi K., Otera H. and Mihara K. (2006). Cytosolic factor- and TOM-independent import of C-tail-anchored mitochondrial outer membrane proteins. *EMBO J.* 25, 5635-5647. 10.1038/sj.emboj.7601438 17110923 PMC1698885

[JCS200204C67] Shamseldin H. E., Alshammari M., Al-Sheddi T., Salih M. A., Alkhalidi H., Kentab A., Repetto G. M., Hashem M. and Alkuraya F. S. (2012). Genomic analysis of mitochondrial diseases in a consanguineous population reveals novel candidate disease genes. *J. Med. Genet.* 49, 234-241. 10.1136/jmedgenet-2012-100836 22499341

[JCS200204C68] Shigemitsu S., Cao W., Terada T. and Shimizu K. (2016). Development of a prediction system for tail-anchored proteins. *BMC Bioinformatics* 17, 378 10.1186/s12859-016-1202-7 27634135 PMC5025589

[JCS200204C69] Studier F. W. (2005). Protein production by auto-induction in high density shaking cultures. *Protein Expr. Purif.* 41, 207-234. 10.1016/j.pep.2005.01.016 15915565

[JCS200204C70] Vilardi F. and Lorenz H. (2011). WRB is the receptor for TRC40/Asna1-mediated insertion of tail-anchored proteins into the ER membrane. *J. Cell Sci.* 40, 1301-1307. 10.1242/jcs.084277 PMC3115773 21444755

[JCS200204C71] Vogl C., Panou I., Yamanbaeva G., Wichmann C., Mangosing S. J., Vilardi F., Indzhykulian A. A., Pangršič T., Santarelli R., Rodriguez-Ballesteros M. et al. (2016). Tryptophan-rich basic protein (WRB) mediates insertion of the tail-anchored protein otoferlin and is required for hair cell exocytosis and hearing. *EMBO J.* 31, 17-31. 10.15252/embj.201593565 PMC5283584 27458190

[JCS200204C73] Wang F., Brown E. C., Mak G., Zhuang J. and Denic V. (2010). A chaperone cascade sorts proteins for posttranslational membrane insertion into the endoplasmic reticulum. *Mol. Cell* 40, 159-171. 10.1016/j.molcel.2010.08.038 20850366 PMC3652556

[JCS200204C75] Wiese S., Gronemeyer T., Ofman R., Kunze M., Grou C. P., Almeida J. A., Eisenacher M., Stephan C., Hayen H., Schollenberger L. et al. (2007). Proteomics characterization of mouse kidney peroxisomes by tandem mass spectrometry and protein correlation profiling. *Mol. Cell. Proteomics* 6, 2045-2057. 10.1074/mcp.M700169-MCP200 17768142

[JCS200204C76] Yagita Y., Hiromasa T. and Fujiki Y. (2013). Tail-anchored PEX26 targets peroxisomes via a PEX19-dependent and TRC40-independent class I pathway. *J. Cell Biol.* 200, 651-666. 10.1083/jcb.201211077 23460677 PMC3587837

[JCS200204C77] Yamamoto Y. and Sakisaka T. (2012). Molecular machinery for insertion of tail-anchored membrane proteins into the endoplasmic reticulum membrane in mammalian cells. *Mol. Cell* 48, 1-11. 10.1016/j.molcel.2012.08.028 23041287

